# Birth of a Social Mediatrician: Adopting Slack, Twitter, and Instagram for Residents

**DOI:** 10.7759/cureus.32569

**Published:** 2022-12-15

**Authors:** July Lee, Bradford Nguyen, Samantha Scanlon, Caroline Rassbach

**Affiliations:** 1 Department of Pediatrics, Stanford University School of Medicine, Lucile Packard Children's Hospital, Palo Alto, USA

**Keywords:** covid-19 pandemic, residency program, connectedness, recruitment, medical education, social media

## Abstract

Introduction

Physicians have increasingly used social media platforms to review new research, expand networks, and communicate. However, few studies have evaluated how the integration of social media into residency programs affects training. This is relevant during the COVID-19 pandemic, with a shift towards virtual formats for medical education, community building, and recruitment.

Objective

The objective of this study was to evaluate how the integration of social media platforms, including Slack, Twitter, and Instagram, influences education, social connectedness, and recruitment within a residency program.

Methods

In 2020, pediatric residents at one institution were encouraged to create personal Twitter and Instagram accounts if they did not already have one and follow the residency program's Twitter and Instagram accounts. Residents were also encouraged to enroll in a private Slack network within the residency program. We surveyed residents in May and June 2020 (pre-intervention) and March 2021 (post-intervention). Analytics from the residency program's social media accounts and Slack were recorded. Data were analyzed using a mixed-methods approach.

Results

Response rates from residents regarding the impact of social media interventions on education, connectedness, and recruitment were 98% (100/102) pre-intervention and 74.5% (76/102) post-intervention. During the study period from May 2020 to March 2021, chief resident posts on the residency program's Twitter and Instagram accounts garnered 447,467 and 151,341 impressions, respectively. Posts with the highest average impressions were those related to advocacy. After the intervention, residents reported increased connectedness to residents in other classes and increased usage of their personal Twitter and Slack accounts for learning and education. Residents rated the program's Instagram account as a useful recruitment tool. Feasibility of posting was assessed by the number of posts by chief residents during the study period (Twitter n=806, Instagram n=67). There were no costs.

Conclusion

Our data shows that social media in residency is feasible, cost-effective, and valuable for education, connectedness, and recruitment. We outlined specific ways social media was feasible and useful in these domains.

## Introduction

Even before COVID-19, communication in training programs was challenging. Residents use many modes of communication that include emails, paging, and smartphone-enabled applications such as Voalte, WhatsApp, iMessage, and Epic Secure Chat. Over time, the best use of technology will continue to evolve as current literature shows that social media's effect on residency education, recruitment, and professionalism is mixed [1-5]. The risks and benefits of various communication modalities to education and connectedness must be evaluated as residency programs grow and adapt to new technologies [1-9]. Slack, a closed communication platform, is effective for communication within the research community and in medical education; it has not yet been explored as a modality for streamlined communication, team collaboration, dissemination of information, file management, and integration of asynchronous curricula in our residency program [10-13]. 

Social media platforms such as Twitter, a public microblog social media community, have been increasingly utilized among various medical professions to gain exposure to new research, expand networks, increase engagement, and disseminate information [14-17]. During the COVID-19 pandemic, an enhanced social media presence has been observed in residency training across the US [18-20]. Social media is expanding in residency training as a means of virtual recruitment during this COVID-19 pandemic, and more than 34% of pediatric residency social media accounts on Twitter, Instagram (a photo and video sharing platform), and Facebook (a social network) were started after March 1, 2020, when the pandemic was in its early stages [18].

Social connectedness is a concept that can be useful in exploring the experience of belonging and relatedness between people [21]. This study aims to understand how integrating Slack, Twitter, and Instagram into a residency program influences education and connectedness. We will be assessing resident perception and their use of social media before and after the formal integration of social media.

## Materials and methods

Study design

From May to June 2020 during the COVID-19 pandemic, all 2020-2021 residents in the Stanford University pediatric residency program (Stanford, California) were enrolled in our residency-specific Slack account, which was sponsored by the University. Residents were also encouraged to create a personal Twitter and Instagram account if they did not already have one in an effort to study if there was a perceived improvement in communication, connectedness, and education. Education and training on these platforms were provided by the chief residents for all the residents during a class-wide retreat. Slack was introduced as a workplace communication platform for announcements, postings, and sharing of educational materials. Chief residents posted on Slack using their individual accounts. The residency program's Twitter account was used for educational tweetorials (tutorials that summarized materials from conferences), advocacy, and recognition of others. The residency program's Instagram account was included after the pre-survey was administered and was used to assess social connectedness and virtual recruitment through posts related to advocacy, recognition of others, featuring residents in a series called "Behind-the-Mask", and highlighting rotations and social events. Chief residents posted collectively as a group on the residency program's Twitter and Instagram accounts. All posts occurred as part of chief residents' communication, teaching, and recruitment responsibilities. 

Data collection and analysis

Data were collected using a mixed-methods approach, including surveys with Likert-scale items and open-ended responses and data from social media. Social media data included metrics such as impressions (times a post was seen or crossed an individual's screen) and followers (number of users that have subscribed to receive updates on a social media platform). This data was pulled from analytics provided by the social media sites respectively. 

We administered a pre-intervention survey using Qualtrics (Qualtrics, Provo, Utah) to rising second-year and third-year residents in May 2020 and incoming interns in June 2020. A post-intervention survey was administered to all three aforementioned classes in March 2021. The survey assessed residents' sense of connectedness to one another and to the residency program and their use of and perceptions of social media platforms. Residents also provided anonymized free-text responses to evaluate the effect of social media on their residency experience. Sense of connectedness questions were adapted from previously published research, and additional questions were created de novo to address study objectives [21]. Incoming interns additionally received a separate post-match survey in June 2020 to assess if they ever visited the residency program's social media accounts during recruitment season and their usefulness in learning more about the residency program.

Data were analyzed using the Wilcoxon signed-rank test, a nonparametric test, to compare pre- and post-responses. Feasibility was measured by the number of chief residents' posts during the study period. All three chief residents shared equal responsibility in creating posts. This study was deemed exempt by the Stanford Institutional Review Board.

## Results

There were 100/102 (98%) respondents for the pre-intervention survey and 76/102 (75%) respondents for the post-intervention survey. Of these respondents, 67 pre- and post-surveys were pairable for Wilcoxon signed-rank test analysis. We received pre-intervention responses from rising second-year (n=33) and third-year residents (n=32) in May 2020 and incoming interns (n=35) in June 2020, and post-intervention responses from all three aforementioned classes (n=76) in March 2021.

Social media usage

As of March 2021, the Slack network had an average of 67 weekly active users, the residency program's Twitter account had 615 followers, and the residency program's Instagram account had 1297 followers. 

From May 2020 to March 2021, the residency program's Twitter posts (n=806) garnered 447,467 impressions, and the residency program's Instagram posts (n=67) garnered 151,341 impressions. Posts with the highest number of impressions were those related to advocacy (i.e., posts about a White Coats For Black Lives rally, a resident-led rally related to the COVID-19 vaccine rollout, encouraging masking, and promoting voter registration) followed by video posts (i.e., videos about recruitment, advocacy needs, and resident life). For example, the highest number of views for a single tweet was 26,260 (18 retweets, 121 likes), which was related to advocacy for the COVID-19 vaccine rollout. 

Social media for education

**Figure 1:**
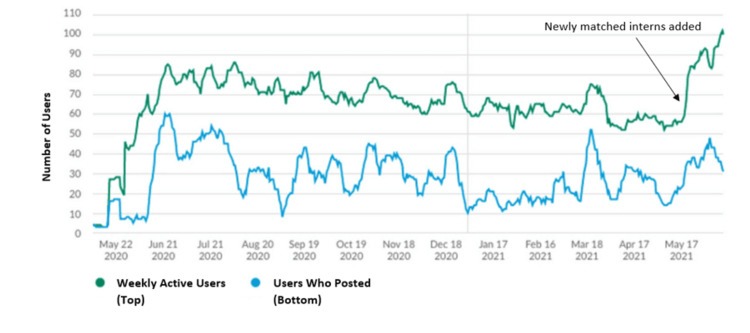
Residents' usage of Slack from May 2020 to May 2021

For residents' self-reported usage of Slack and Twitter for learning, the Wilcoxon signed-rank test supported the rejection of the null hypothesis (p-value <0.0001 and 0.006, respectively), thus there is a significant increase in resident use of both Slack and Twitter in the context of learning (Table 1). Analytics showed a fluctuation in usage within Slack throughout the year (Figure 1). Slack was noted to be "an effective way to share the knowledge that is often given in 1:1 sessions with the entire residency. It also serves as a form of institutional memory". Residents commented on their appreciation for how Slack could store educational materials in a searchable format. Residents also felt that the residency program's Twitter was useful for education, especially "the educational Twitter threads", "mini-quizzes", "reference for morning reports that we do not get to attend", and "updates about papers and other cool things that people in my program published or accomplished". Each tweetorial received an average of 897 impressions.

**Table 1:** Descriptive statistics and Wilcoxon signed-rank test comparison of pre- and post-responses for: "In the context of learning, how often do you use each of the following?" (N=67) 1: never, 2: ≤ monthly, 3: few times/month, 4: few times/week, 5: daily

	Pre mean	Post mean	Pre median (Q1-Q3)	Post median (Q1-Q3)	Wilcoxon, N	Wilcoxon p-value
Email	3.95	3.97	4 (3-5)	4 (3-5)	44	0.9681
Texting	3.23	3.17	3 (2-4)	3 (2-4)	46	0.7718
WhatsApp	2.21	2.03	2 (1-3)	2 (1-3)	33	0.3173
Slack	1.68	2.68	1 (1-2)	3 (2-4)	48	<0.0001
Twitter	1.66	2.27	1 (1-2)	2 (1-3)	36	0.0006
Facebook	1.62	1.44	1 (1-2)	1 (1-2)	29	0.2187
Medical journals	3.34	3.25	3 (3-4)	3 (3-4)	31	0.3843

Social media and connectedness

After the intervention, we found a statistically significant increase in the mean connectedness to residents in other classes (Table 2). There were no significant differences in connectedness to other residents within a class or to the residency program. 

**Table 2:** Resident connectedness before and after social media intervention (N=67) 1: Not at all, 2: slightly 3: somewhat, 4: very, 5: extremely

Resident connectedness to the residency program before and after the social media intervention						
	Pre mean	Post mean	Pre median (IQR1-IQR3)	Post median (IQR1-IQR3)	Wilcoxon, N	p-value
All years	3.30	3.24	3 (3-4)	3 (3-4)	41	0.6171
PGY3+ (N=21)	3.29	3.48	3 (3-4)	4 (3-4)	12	0.3898
PGY2 (N=19)	3.26	3.47	3 (3-4)	4 (3-4)	12	0.3681
PGY1 (N=27)	3.33	2.89	3 (3-4)	3 (2.5-3)	17	0.0549
Resident connectedness to other residents in their class before and after the social media intervention						
	Pre mean	Post mean	Pre median (IQR1-IQR3)	Post median (IQR1-IQR3)	Wilcoxon, N=	P-Value
All years	3.67	3.61	4 (3-4.5)	4 (3-4)	34	0.6455
PGY3+ (N=21)	4.00	3.86	4 (3-5)	4 (4-4)	10	0.4473
PGY2 (N=19)	4.16	3.95	4 (3.5-5)	4 (3-4.5)	9	Insufficient N
PGY1 (N=27)	3.07	3.19	3 (3-3)	3 (3-4)	15	0.5687
Resident connectedness to residents in other classes before and after the social media intervention						
	Pre mean	Post mean	Pre median (IQR1-IQR3)	Post median (IQR1-IQR3)	Wilcoxon, N=	P-Value
All years	2.28	2.67	2 (2-3)	3 (2-3)	36	0.0021
PGY3+ (N=21)	2.29	2.81	2 (2-3)	3 (2-3)	12	0.0278
PGY2 (N=19)	2.79	2.89	3 (2.5-3)	3 (2.5-3)	10	0.5755
PGY1 (N=27)	1.93	2.41	2 (1-3)	3 (2-3)	14	0.0238

On average, residents agreed that Slack and the residency program's Instagram were useful tools for fostering connectedness, while they felt the residency program's Twitter was less useful for this purpose (Table 3). Some residents reported important perceptions: "Slack has allowed us to communicate between classes more! And to be up to date with current events or notifications happening inside the residency program", and "[Instagram] has created a sense of 'belonging' and makes me feel more connected to the rest of the program".

**Table 3:** Slack, Twitter, Instagram, and connectedness after social media intervention 1: completely disagree, 2: disagree, 3: somewhat disagree, 4: neither agree or disagree, 5: somewhat agree, 6: agree, 7: completely agree

Slack, Twitter, Instagram, and connectedness	Post-intervention mean (SD)
Slack allowed me to better connect with my residency program	
All years (N=76)	4.51 (1.80)
PGY1 (N=28)	4.61 (2.02)
PGY2 (N=25)	4.24 (1.81)
PGY3+ (N=23)	4.70 (1.52)
Twitter allowed me to better connect with my residency program	
All years (N=76)	3.29 (1.71)
PGY1 (N=28)	3.14 (1.65)
PGY2 (N=25)	3.56 (1.66)
PGY3+ (N=23)	3.17 (1.87)
Instagram allowed me to better connect with my residency program	
All years (N=76)	4.43
PGY1 (N=28)	3.96
PGY2 (N=25)	4.6
PGY3+ (N=23)	4.83

While responses were primarily positive, a minority of residents shared negative experiences: "[I] had a hard time getting in the habit of using [Slack] and often miss information", and "there were occasions the program Instagram made me feel disconnected from the program because the positive nature of the posts did not match my personal experience".

Social media and recruitment

We used Instagram largely for recruitment. On average, residents agreed (mean 5.22/7) that the residency program's Instagram account was a useful tool for recruitment (Table 4).

**Table 4:** Instagram as a tool for recruitment 1: completely disagree, 2: disagree, 3: somewhat disagree, 4: neither agree or disagree, 5: somewhat agree, 6: agree, 7: completely agree

Instagram as a tool for communication with applicants	Post-intervention mean (SD)
The StanfordPeds Instagram profile is a useful tool for communicating with applicants.	
All years (N=75)	5.22 (1.58)
PGY1 (N=28)	5.48 (1.33)
PGY2 (N=25)	5.17 (1.30)
PGY3+ (N=22)	5.22 (1.58)

In a separate post-match survey, 86% (n=31) of our newly matched interns starting June 2021 viewed our residency program's Instagram account during recruitment season and reported that it was extremely useful (4.7/5). The most useful posts were the "Behind-the-Mask" series which spotlighted residents for applicants and other residents. Other helpful series included videos that showcased "A-day-in-the-life" of the residents in different rotations, Instagram live streams that connected applicants with the residents, and posts that showcased the residency program leaders. 

## Discussion

We report here a social media intervention to bring Slack, Twitter, and Instagram to our pediatric residency program. The COVID-19 pandemic encouraged a need for virtual tools in the areas of medical education, communication, and recruitment. While some results of our interventions were expected based on the common use of social media platforms, we were also surprised that residents and applicants utilized these tools in new and unexpected ways. Slack was used to promote connectedness and sharing resources internally. Twitter was perceived as effective for education, while Instagram was most appreciated for recruitment and connectedness. These findings align with prior research showing that social media supports education and recruitment [22-24].

Social media for education

Social media is increasingly used in medical education as a platform to share research, information, and resources [4, 10, 11, 14, 20, 22, 23, 25-29]. In this educational intervention, Slack initially served as an internal communication platform, but it was quickly recognized by residents as a way to also disseminate and store educational materials. Several residents developed education-focused channels on Slack that created a searchable repository of resources for all residents to access, including channels on "Clinical Resources" and "Electronic Health Record Tips". Much of what is published on Slack in medical education is in regard to coordination, collaboration, and communication [10-13]. Our findings add to the literature by showing new ways to use this platform for asynchronous education.

Twitter was heavily used for educational purposes to allow anyone public access to our educational conferences through our residency program's Twitter account. Tweets that included interactive components such as polls often garnered the most interest. Many residency programs maintain Twitter handles, and many more residency programs created accounts during the 2020-2021 academic year, given the pandemic [18]. Our findings on the role of Twitter in medical education support other studies that have shown that Twitter is increasingly utilized in the area of medical education in general and adds a pediatric residency perspective [4, 22, 25-29]. We have since seen several posts from our residents, including one focused on patient-centered care that reached nearly 150,000 likes.

Social media and connectedness

Social media has long been utilized to network. In this study, Slack and Instagram were platforms used to increase connectedness within our residency program. Slack was often used for quick communications with residency program leadership as well as within and between classes. Examples of how Slack was used to communicate include arranging group events such as pick-up sports or hikes, as well as current/incoming residents creating a shared moving/selling channel at the end of the year.

Instagram provided a means to share personal stories and residency-related experiences publicly using our residency program's Instagram account, allowing residents and applicants to connect virtually. Instagram is increasingly used by residency programs to share content, and our intervention demonstrates novel ways of promoting connectedness. While our data show improved interclass connectedness, we would anticipate that to naturally occur with time in any given year. And although our data did not show changes in resident connectedness to the residency program or to others in their class, this finding could be a reflection of the isolation and separation caused by the COVID-19 pandemic. It is also worth noting that not all residents interacted with social media regularly, and a small number of survey respondents expressed a sense of "fear of missing out" (FOMO) which made them feel less connected. This should be further evaluated as existing literature shows concerns that social media can have impacts on the mental health of individuals [30]. 

Social media and recruitment

Recruitment quickly became a focus of our social media efforts, given the shift toward virtual recruitment during the COVID-19 pandemic. Residents regarded Instagram as a platform for recruitment for its visual appeal, interactivity, and utility in showcasing residents and the culture of the residency program during a time when applicants were not permitted to visit the residency program. Through a variety of recurring series on our residency program's Instagram account, including our "Behind-the-Mask" series, Instagram live Q&As, "A-day-in-the-life," and team features, we were able to showcase residents, hospital teams, and experiences. Instagram also allowed us to reach a broad audience, many of whom were also looking to get to know our residency program as prospective residents. Most who matched had viewed and positively rated the residency program's Instagram account.

Limitations

Our survey data are from a single institution and are based on self-reports, which may not accurately represent the true frequency of social media use. Not all study participants responded to the post-survey, which may have led to nonresponse bias. The effects of COVID-19-related restrictions may have overpowered the results of our social media intervention. Furthermore, our evaluation of Instagram was limited due to its non-inclusion in the pre-survey. Social media analytics are limited in that it is impacted by any viewing of a post, including by the study designers, and do not necessarily imply a post was read, only that it appeared on a screen. We are also unable to assess the source of impressions, which may be influenced by several factors, including retweets, bots (profiles not controlled by a person), and other unmeasurable unknowns. 

## Conclusions

Overall we report a social media intervention in our residency program that included Slack, Twitter, and Instagram. The COVID-19 pandemic impacted residents during this intervention, and the effect of social media should continue to be evaluated outside of a global pandemic. This was a cost-effective intervention that required a shift towards the usage of newer and more interactive social media platforms that have the potential to reach wider audiences, as evidenced by nearly 600,000 cumulative impressions from our social media platforms. A longer-term evaluation of a social media intervention could be helpful as more trainees and educational leaders become engaged in social media, and there is an increased use of these platforms in future virtual recruitment seasons. The negative comments and sentiments around social media should be further explored to better understand the risks. Further studies should also assess the knowledge retention of residents using social media interventions. Nonetheless, our findings demonstrate the potential of social media to impact education, connectedness, and recruitment, and we outlined specific ways social media was feasible and useful in these domains.
